# Unusual Case of Gallbladder Adenocarcinoma Metastasis to the Abdominal Wall 11 Years Later: Synchronous Presentation with Two Malignant Colon Tumors, Coincidence or Not?

**DOI:** 10.1155/2021/6662275

**Published:** 2021-02-26

**Authors:** Belén Matías-García, Fernando Mendoza-Moreno, Manuel Díez-Alonso, Ana Quiroga-Valcárcel, Elena Aguirregoicoa-García, Cristina Vera-Mansilla, Enrique Ovejero-Merino, Javier Mínguez-García, Diego Córdova-García, Alberto Gutiérrez-Calvo

**Affiliations:** ^1^Department of General and Digestive Surgery, Príncipe de Asturias Teaching Hospital, Alcalá de Henares, Madrid, Spain; ^2^Department of Pathology, Príncipe de Asturias Teaching Hospital, Alcalá de Henares, Madrid, Spain

## Abstract

**Introduction:**

Abdominal wall masses are a common finding in clinical practice. A high percentage of these masses are malignant. We present the case of a patient operated for a gallbladder adenocarcinoma, who consulted eleven years later for a malignant mass of the abdominal wall in synchrony with two adenocarcinomas of the left colon and sigmoid. *Case Report*. A 75-year-old male underwent a laparoscopic cholecystectomy with an incidental diagnosis of adenocarcinoma in situ (TisN0M0 according to AJCC 8th edition). The operative report mentioned that the removal of the gallbladder was difficult due to the inflammatory process, and the gallbladder was accidentally opened during the operation. It was not clear from the operative report whether an extraction bag was utilized to remove the specimen, but the histopathological study confirmed an open gallbladder. He presented 11 years later with an asymptomatic heterogeneous complex cystic mass involving the anterior rectus abdominis muscle. Colonoscopy showed synchronous tumors in the descending and sigmoid colon with pathology confirming adenocarcinoma. The patient underwent an elective laparotomy with resection of the anterior abdominal wall mass, left hemicolectomy, and sigmoidectomy. The histopathological results of the abdominal mass (CK7, CK20, EMA, CEA positive) were described as metastasis of adenocarcinoma of biliary origin. *Discussion*. Port site recurrences are rare complications following laparoscopic surgery when malignancy is unsuspected. Possible factors related to local implantation include direct seeding of spilled bile or tumor cells into the wound or shedding of tumor cells due to pneumoperitoneum-induced loss of the peritoneal barrier at the trocar site. In the absence of distant metastasis, treatment should include wide port site excision with malignancy-free surgical margins.

**Conclusion:**

Abdominal wall metastasis from gallbladder carcinoma is rare, and its synchronous presentation with a malignant neoplasm of the colon is exceptional. This is the first report of a patient with abdominal wall metastasis from a gallbladder adenocarcinoma operated eleven years ago that debuted synchronously with two adenocarcinomas of the left colon and sigma.

## 1. Introduction

Abdominal wall masses are a common finding in clinical practice and up to 42.2% can be malignant [1]. The patient's history, physical examination, and imaging tests play an important role in the differential diagnosis. However, in most cases, the definitive diagnosis is made pathologically following surgical excision.

We present the first report of a patient with a metastatic mass in the abdominal wall from a gallbladder adenocarcinoma operated on eleven years earlier by laparoscopic cholecystectomy, who presented with a synchronous sigmoid and descending colon adenocarcinoma.

Laparoscopic cholecystectomy is the standard operation for symptomatic cholelithiasis and other benign gallbladder diseases [2, 3]. The increase in the number of laparoscopic surgical procedures has led to the discovery of an increased number of incidental gallbladder carcinomas at an early stage [2]. Histopathological examination reveals an incidental carcinoma in approximately 1-2% of patients undergoing laparoscopic cholecystectomy [3–5].

Moreover, the association between gallbladder polyps and adenomatous colon polyps is well known and will be discussed.

## 2. Case Report

**Figure 1:**
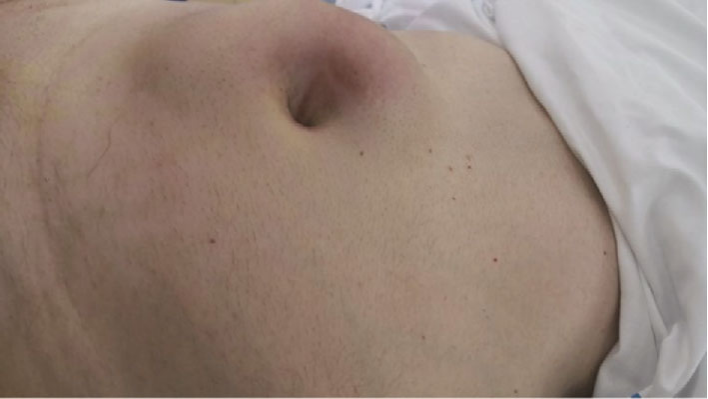
Asymptomatic mass of 12 × 6 cm (CC × AP) in the mesogastrium with petrous consistency.

A 75-year-old Caucasian male presented in September 2019 with a long-term asymptomatic mass (for approximately the last 3 years) that had increased in recent years (Figure 1).

The patient had a medical history of laparoscopic cholecystectomy for cholelithiasis in 2008. The pathological study indicated a chronic cholecystitis with an incidental diagnosis of adenocarcinoma in situ over a 12 mm adenomatous polyp located at the bottom. The operative report mentioned that the removal of the gallbladder was difficult due to the inflammatory process, and the gallbladder was accidentally opened during the operation. It was not clear from the operative report whether an extraction bag was utilized to remove the specimen, but the histopathological study confirmed an open gallbladder. The patient did not undergo any adjuvant therapies, and there was no postoperative follow-up medical history.

**Figure 2:**
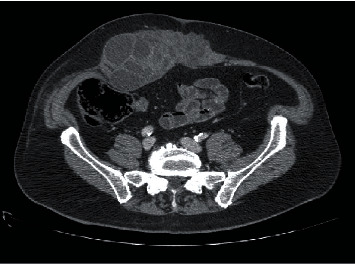
Heterogeneous mass in the abdominal wall that affects the rectus abdominis muscle, predominantly cystic with septa inside.

**Figure 3:**
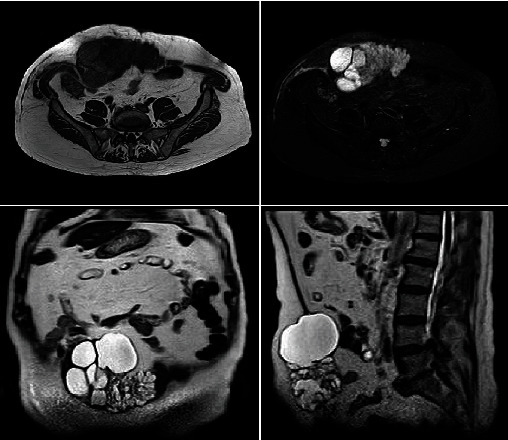
Nodular lesion of cystic predominance, multilocated, hyperintense in T1 and hypointense in T2, which affects the rectus abdominis muscle, exceeding the midline.

The current physical examination revealed a mass effect in the mesogastrium of stony consistency, painless to palpation and no signs of peritonitis. An abdominal contrast-enhanced computed tomography (CT) scan was performed (Figure 2). A heterogeneous mass in the abdominal wall measuring 12 × 6 cm, predominantly cystic, with septa inside, was noted affecting the rectus abdominis muscle. A subsequent magnetic resonance imaging (MRI) further delineated the mass lesion with no associated abnormality in the rest of the biliary system or pancreas (Figure 3).

Given the presence of mucin and the history of adenocarcinoma in situ of a gallbladder polyp, a gastroscopy and a colonoscopy were performed. The colonoscopy showed tumors in the descending colon and sigma with biopsies from both sites confirming adenocarcinoma.

**Figure 4:**
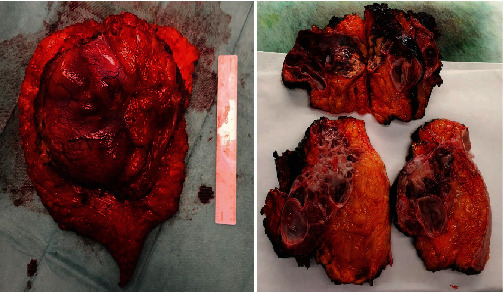
Well-defined mass with septa inside, which is of interest to the skin, fatty tissue, muscle, and the posterior sheath of the anterior rectus abdominis without affecting the peritoneum or abdominal organs.

The patient underwent an elective laparotomy. We found an amorphous, well-defined mass that involved the skin, subcutaneous fat, the rectus muscle, and the posterior sheath of the rectus without affecting the peritoneum or the abdominal organs (Figure 4). En bloc resection with surgical margins (R0 resection) free of tumor was performed. After that, the abdominal cavity was explored without any suspicion of malignancy, liver metastasis, or peritoneal carcinomatosis. Then, a left radical colectomy and sigmoidectomy were performed. Finally, we repaired the defect in the abdominal wall using a prosthetic mesh (GoreTex Dual Mesh, Bard®) as a bridge between the two aponeuroses. The postoperative period was uneventful. The patient was discharged on the seventh day after surgery.

**Figure 5:**
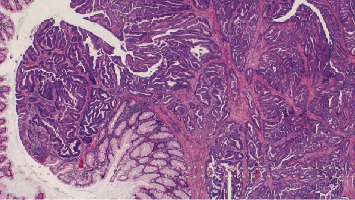
Left hemicolectomy. Moderately differentiated adenocarcinoma of the large intestine.

The histopathological results were described as metastasis of adenocarcinoma of biliary origin (positive for CK7, CK18, CK19, CK20 (focal), CDX2 (focal), MUC1, MUC5AC, MUC2 (weak and focal), EMA, CA19. 9, CEA (focal), CD10 (focal), beta-catenin; negative for CA125, PAX8, PSA, TTF1, S100, estrogen and progesterone receptors, CD34, inhibition) without affecting surgical margins. The histological study of the colon revealed an adenocarcinoma in sigma T3N0M0 (according to the eighth edition of the AJCC) while the left colon showed an adenocarcinoma in situ (TisN0M0) (Figure 5). The patient was evaluated by the oncology department, and no adjuvant therapy was recommended.

During the follow-up (18 months), the patient has not presented evidence of recurrence, wound infection, or mesh rejection. The follow-up schedule based on an abdominal contrast-enhanced computed tomography scan and analytical blood test with tumor markers (CEA and CA 19.9) was every 6 months.

## 3. Discussion

Abdominal wall masses are a frequent finding in clinical practice and cover a large number of pathologies. Abdominal masses are traditionally classified as benign tumors, malignant tumors, or tumor-like lesions such as hernias or abscesses of the abdominal wall [6]. According to a cohort study, malignant masses accounted for 42.2% of these [1]. Within the malignant lesions, we can differentiate between primary and secondary tumors.

The patient's clinical history and personal history play a very important role in the diagnostic suspicion of abdominal masses. It is well known that benign lesions tend to grow slowly, with a long time of evolution. On the other hand, malignant lesions tend to have a more aggressive course with a faster growth of the tumor mass. In our patient, the antecedent of interest was the accidental finding of an adenocarcinoma in situ of the gallbladder. Eleven years later, the patient consulted for a tumor in the abdominal wall that had progressively grown after gallbladder surgery and was not causing symptoms. Despite the long time since the gallbladder surgery, the patient started to notice the abdominal wall mass 3 years ago. Since then, it grew slowly, with minimal local discomfort. Given the long time of development of the tumor, the probability that the mass was secondary to gallbladder adenocarcinoma was initially low. However, the surgical wound of the umbilical port was engulfed by the tumor mass.

In relation to imaging tests, ultrasound is usually a good imaging technique for the initial diagnosis because it is cheap and not harmful to the patient. Furthermore, most abdominal masses are superficial and therefore accessible by ultrasound [6]. However, it is operator dependent and its use is limited in large and deep masses [6]. Contrast-enhanced cross-sectional imaging (CT or MRI) represent the mainstays of initial diagnosis and work-up, providing information regarding relationship with structures and possible primary site and characterizing the tumor. Consecutive improvements in imaging techniques such as CT and MRI have made their use popular as diagnostic tests. MRI is widely considered the optimal imaging technique in the evaluation of soft tissue tumors because of its high resolution [6]. CT has less of an advantage in diagnosing the aetiology of abdominal wall masses [6]. However, it can be used to study relationships with adjacent structures and diagnose the primary tumor or even distant site metastases. The set of imaging tests guides us towards the definitive diagnosis based on the characteristics of the tumor (size, location, and relationship with neighbouring structures) and its component (liquid, fat, blood, etc.).

However, some abdominal wall masses remain undetermined due to the heterogeneous spectrum of disease and the limited capability of imaging tests [6]. While imaging can provide preoperative information for surgical planning, the diagnosis requires histopathological examination typically following surgical resection. In our patient's case, the pathological anatomy of the abdominal wall tumor described the fatty and muscular tissue in which neoplastic proliferation is arranged. This neoplastic proliferation was composed of cystic glandular structures covered by a layer of cubic or columnar cells with abundant apical mucin, with formation of micropapillae and occasional cribriform structures, with mild-moderate nuclear atypia. They were surrounded by fibrocollagen tissue and were accompanied by haemorrhagic and cystic degenerative changes, foamy accumulations of histiocytes, fibrosis, and steatonecrosis. This description would be compatible with the original neoplasm in the biliary tract. However, the immunohistochemical profile of the piece was also analysed. Cytokeratins (CK), carcinoembryonic antigen (CEA), and epithelial membrane antigen (EMA) have been well related in the literature as useful markers in the diagnosis of carcinoma of biliary origin [7, 8]. In our patient's case, the surgical part of the abdominal tumor was positive for CK types 7, 18, 19, 20, EMA, and CEA. Within cytokeratins, the combination of monoclonal antibodies CK20 and CK7 has been useful to discriminate primary and metastatic tumors. In a previous autopsy-based study of liver metastases, it was found that the CK20+/CK7- phenotype of liver metastasis indicated a 78% probability that the primary tumor was located in the colon or rectum, while the CK20+/CK7+ phenotype was associated with a 74% probability of pancreatobiliary origin of the metastasis [9, 10]. When gastric metastases were excluded, these statistical probabilities increased to 94% and 92%, respectively [9, 10]. The positivity of the piece for both antibodies leads us to a metastasis of pancreatobiliary origin and would exclude the colorectal origin. The flow-related homeobox transcription factor (CDX-2) regulates the differentiation of intestinal epithelial cells [11]. Strong nuclear staining for CDX-2 is always present in the epithelial cells of the small intestine and colon [11]. However, scattered gallbladder epithelial cells and ductal and acinar cells from the pancreas can also be CDX-2 positive [11]. In our patient's case, the nuclear stain for CDX-2 was focal positive, leading us to a biliary origin rather than a colorectal origin. Approximately 90% of colorectal carcinoma cases express nuclear B-catenin [11]. The adenomatous polyposis coli (APC) mutation leads to nuclear accumulation of B-catenin, a characteristic associated with the progression along the sequence of the adenoma-carcinoma [11]. Therefore, the presence of B-catenin would guide us towards the colorectal origin of our patient's abdominal mass. In relation to the expression of mucin markers (MUC), MUC2 can be positive in tumors of the upper and lower gastrointestinal tract, while MUC1 can be positive in upper gastrointestinal tumors but not in lower ones [11]. Our patient's abdominal mass was positive for MUC1 and MUC2. MUC6 is expressed in pancreatic, ampular, and gastric carcinomas, but colon carcinoma is usually negative for MUC6 [11]. In our patient, MUC6 was not tested. Therefore, we assume that the abdominal wall tumor has a higher probability of being secondary to an adenocarcinoma in situ of biliary origin than to an adenocarcinoma of colorectal origin.

**Figure 6:**
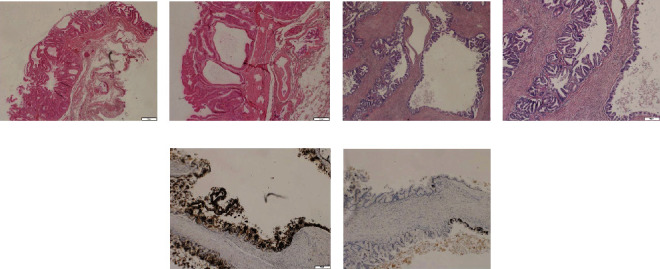
(a, b) Gallbladder with foci of epithelial dysplasia and carcinoma in situ. (c–f) Epithelial neoplastic proliferation composed of cystic structures lined by a layer of columnar or cubic cells, with formation of micropapillae and mild-moderate atypia. In the immunohistochemical study, these cells express CK7 diffusely and CK20 focally.

With the previous results, we reviewed the anatomopathological report of the gallbladder and the histological preparations (Figure 6). The pathological report described an open and fragmented cholecystectomy of approximately 10 × 5 cm, with a solution of continuity and a vegetative lesion of 2 cm whose diagnosis was an adenomatous polyp with foci of severe epithelial dysplasia/adenocarcinoma in situ. They also describe the presence of cholelithiasis. According to the pathology report, the gallbladder was fragmented and therefore it cannot be categorically stated that it was an adenocarcinoma in situ, and similarly, the margins of resection could not be assessed.

Therefore, assuming the diagnosis of secondary metastasis to gallbladder adenocarcinoma and taking into account its location in the mesogastrium, the initial suspicion was implantation at the site of the laparoscopic port. There are two hypotheses about the main factors that may intervene in the mechanism of recurrence of the abdominal wall at the site of the port: one is the systemic progression of the malignancy and the other is local implantation at the port site [12]. In favour of recurrence at the port site as a consequence of systemic progression, there are studies that report cases of patients operated by open cholecystectomy and others by laparoscopic cholecystectomy for incidental disease confined to the mucosa where the intact gallbladder is removed and without bile leakage [13].

Possible factors related to local implantation include direct seeding of spilled bile or tumor cell in the wound or detachment of the tumor cell due to loss of the peritoneal barrier at the trocar site and induced by the pneumoperitoneum in the peritoneal cavity [2, 14]. The possible involvement of the pneumoperitoneum in the pathogenesis can be explained by the turbulent flow of gas at the moment of deflation of the pneumoperitoneum, mainly due to the pressure gradient (the so-called chimney phenomenon) [3, 15]. The continuous passage of instruments to and from the port sites after dissection can lead to direct seeding of the port sites with tumor cells [3, 14].

Wound or port metastasis is a rare but recognized complication in patients undergoing laparoscopic surgery when a malignancy is diagnosed or not suspected [14]. Port site metastasis was first described in 1978 by Döbrönte et al. [16] in a patient who underwent laparoscopy for ovarian cancer (14). Since then, there have been numerous reports of injuries or metastases at port sites in patients with other malignancies. The incidence of port metastases due to gallbladder carcinoma varies between 10% and 29% in the literature [2, 14]. It makes no difference whether the tumor is confined to the gallbladder (T1/T2) or locally advanced (T3/T4) [15].

The average time of recurrence at the port site after cholecystectomy reported in the literature is 7 months [2, 17]. Recurrence in the abdominal wall within a few months after cholecystectomy implies that it is a manifestation of the aggressive behaviour of gallbladder cancer [2]. However, some cases have been reported where the first sign of recurrence at the port site appeared years after cholecystectomy [2, 18]. Our patient consulted for an abdominal wall tumor 11 years after cholecystectomy. This is the only case reported with a time interval to recurrence greater than 3 years. The probable reason for the slow growth of the recurrent tumor could be the early stage of gallbladder cancer.

However, since laparoscopic surgery is currently fully implemented, port metastasis can be reduced by meticulous surgical resection to avoid cell or bile leakage [14]. In addition, the use of surgical specimen removal bags or wound protectors is reported to reduce the incidence of port metastases [14]. In expert hands, the risk of seeding malignant cells during laparoscopic cholecystectomy may be even lower than in open surgery (14). However, Nakagawa et al. recommend excision of the port sites during laparoscopic cholecystectomy if the gallbladder wall is perforated during dissection (2).

Treatment will depend on the presence of disseminated disease. In the absence of distant metastasis, treatment should include wide port site excision with malignancy-free surgical margins (R0 resection) and exploration of the peritoneal cavity to exclude peritoneal metastasis (14). However, it is not clear whether the presence of wound or port site metastases can be considered a risk factor for peritoneal spread of disease [14] or whether it should be considered a disseminated disease [19]. Although the presence of long-term survivors following resection of metastases may suggest that in some cases, they represent an isolated recurrence [19].

The prognosis in patients who have developed wound or port site metastases for gallbladder carcinoma is unclear [2]. There are reported cases with recurrence at the port site with a body-wide prognosis [3, 20]. However, other patients have a long disease-free survival following surgical resection of the metastases [12, 18, 20]. It is possible that these differences are due to a low incidence of pathology, leading to a lack of studies and therefore differences in management.

Furthermore, despite recent advances in diagnostic imaging, initial imaging studies often do not reveal a diagnosis of gallbladder cancer [21]. The clinical presentations of gallbladder cancer in its early stages are nonspecific and the symptoms are often similar to those of gallstone disease [21, 22]. Therefore, patients mistakenly proceed with a simple cholecystectomy as the first surgical procedure [21]. Approximately 15–30% of gallbladder carcinomas are detected incidentally on microscopic examination of samples [16].

The decision to undergo additional treatment in patients with an incidental diagnosis of gallbladder carcinoma should be based on a pathological examination of the resected gallbladder. Previous reports have indicated that simple cholecystectomy and observation is appropriate for patients with pTis or pT1a disease (AJCC 8th ed.) [2]. Cholecystectomy and resection of the segment IVb/V liver, lymphadenectomy of the hepatic hilum, common hepatic artery, and retroduodenopancreatic lymphadenectomy are the standard procedures for pT2 or advanced carcinomas [2, 21]. However, the surgical management of patients with stage T1b gallbladder cancer is still controversially discussed [21], because cholecystectomy alone has a 34% recurrence rate. Furthermore, some authors recommend excision of the port sites at the time of the second resection [23]. In our patient, the histological examination revealed an adenocarcinoma in situ; therefore, no additional interventions were considered necessary.

On the other hand, some studies seem to indicate that the risk of incisional metastasis is higher after laparoscopic cholecystectomy [19, 21]. Therefore, the authors recommend conventional open surgery in cases of known or suspected gallbladder cancer (19, 23).

Finally, the association between gallbladder polyps and adenomatous colon polyps is well known. Several studies suggest that the presence of polyps in the gallbladder is associated with an increased incidence of colorectal adenomas, which in turn can develop into colorectal cancer [24, 25]. In addition, a study of 4626 asymptomatic individuals undergoing ultrasound and colonoscopy suggests that colorectal neoplasia is significantly related to gallbladder polyps, especially those of ≥5 mm [26].

Gallbladder polyps and colorectal neoplasia share several risk factors that could explain their association, such as male gender, age, and metabolic syndrome, including obesity, insulin resistance, and lipid profile abnormalities [25, 26]. In addition, the gallbladder epithelium and colorectal mucosa share some similarities [25, 26]. Furthermore, there are also polyposis syndromes characterized by the presence of gastrointestinal polyps and in other locations such as Lynch syndrome, familial adenomatous polyposis (FAP), MYH-associated polyposis, Cowden's disease, juvenile polyposis, or Peutz-Jeghers syndrome [24]. However, the mechanism underlying the association between gallbladder polyps and colorectal adenomas remains uncertain [25].

However, these studies do not differentiate between cholesterol polyps or adenomatous polyps of the gallbladder. Cholesterol polyps are benign and are the most common [26, 27]. However, adenomatous polyps have malignant potential [27]. Therefore, the association between adenomatous polyps of the gallbladder and adenomatous polyps of the colon is not clear. Therefore, more studies are needed to confirm the association between adenomatous gallbladder polyps and colorectal cancer.

Therefore, we must be consistent with this possible association and additional screening tests such as CT scan or colonoscopy could be justified if polyps are found in the gallbladder. However, more studies are needed to determine this.

## 4. Conclusion

Metastasis in the abdominal wall of a gallbladder carcinoma operated 11 years ago is rare. Its synchronous presentation with two malignant neoplasms of the colon is exceptional and has not been described before. Its treatment is mainly surgical. Its prognosis depends on the tumor stage of the colon and negative surgical margins after resection of the metastasis.

Finally, the association of gallbladder and adenomatous polyps in the colon is well known. Therefore, this association can be taken into account for early detection studies by colonoscopy.
